# Complications of spilled gallstones following laparoscopic cholecystectomy: a case report and literature overview

**DOI:** 10.4076/1752-1947-3-8626

**Published:** 2009-07-24

**Authors:** Sophie Helme, Tushar Samdani, Prakash Sinha

**Affiliations:** 1 Imperial College London, 10th Floor, QEQM Wing, St Mary's Campus, 20 South Wharf Road, London, W2 1PD, UK; 2 Princess Royal University Hospital, Farnborough, Kent, UK

## Abstract

**Introduction:**

Gallbladder perforation is common and occurs in 6 to 40% of laparoscopic cholecystectomy procedures. In up to a third of these cases, stones are not retrieved and complications can arise many years post-operatively. Diagnosis can be difficult and patients may present to many specialties within medicine and surgery. We seek to present our case and review the literature on prevention and management of "lost" stones.

**Case presentation:**

Our patient is a 77-year-old woman who presented to the urology clinic with a loin abscess that developed five years after laparoscopic cholecystectomy. Radiological studies showed retained abdominal gallstones and an associated abscess formation. These were drained under ultrasound guidance on several occasions and the patient now suffers from chronic sinusitis. Due to her age and comorbidities, she has declined definitive surgical intervention to remove the stones.

**Conclusion:**

Gallbladder perforation during laparoscopic cholecystectomy is a reasonably common problem and may result in spilled and lost gallstones. Though uncommon, these stones may lead to early or late complications, which can be a diagnostic challenge and cause significant morbidity to the patient. Clear documentation and patient awareness of lost gallstones is of utmost importance, as this may enable prompt recognition and treatment of any complications.

## Introduction

In the current era of minimally invasive surgery, laparoscopic cholecystectomy has become the gold standard for the surgical treatment of symptomatic gallstones. However, with the increase in the number of laparoscopic operations performed, there has also been a noticeable increase in the number of complications specific to these procedures. Gallstones can be spilled during an open cholecystectomy, but these stones are eliminated usually through direct removal, copious irrigation and mopping with laparotomy sponges. In laparoscopic procedures, these techniques are more difficult or unavailable and so stones can disappear from view and can become "lost". Studies show that the incidence of spilled gallstones during laparoscopic cholecystectomy accounts for 6 to 40% of procedures performed, while 13 to 32% of such operations result in lost stones [1,2]. Complications from stones that are left within the peritoneal cavity can cause unusual but significant morbidity.

## Case presentation

A 77-year-old woman presented to the urology clinic with a two-week history of night sweats, right back pain and loin swelling. Her medical history included a laparoscopic cholecystectomy for gallstones five years before presentation. Other than a similar pain noticed six months previously, there had been no known complications from the surgery. On examination the patient had a tender, fluctuant swelling in the right lumbar region with overlying skin erythema. Her blood tests showed a neutrophilia of 7.7 × 10^9^/litre and C-reactive protein of 134 mg/litre. A computed tomography (CT) scan showed a complex subphrenic, subhepatic and subcutaneous collection. The patient's abscesses were drained under ultrasound guidance and the drains left in situ. The pus grew Escherichia coli on culture. The patient was then treated with antibiotics for ten days and discharged home.

**Figure 1:**
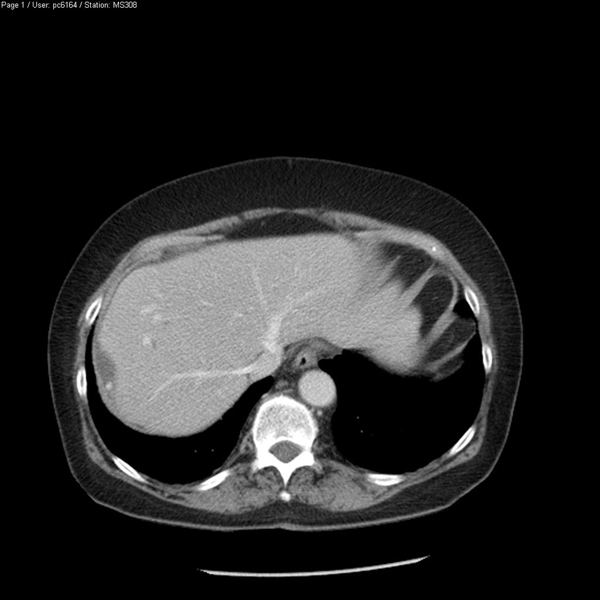
**CT demonstrating perihepatic gallstone**.

Three weeks later the patient reattended hospital with similar symptoms and ultrasound and CT scans showed a perihepatic and subcutaneous reaccumulation of fluid, with a 1cm gallstone adjacent to the right lobe of her liver (Figure 1). The abscesses were again drained. A barium enema of the colon was arranged to exclude a neoplastic cause for the abscess, but the result simply showed mild sigmoid diverticular disease and no fistulous connection. In addition, a contrast study through the percutaneous drain did not reveal any connection with intra-abdominal viscera. Therefore, the patient was diagnosed with intra-abdominal sepsis secondary to retained gallstones at the time of her laparoscopic cholecystectomy.

**Figure 2:**
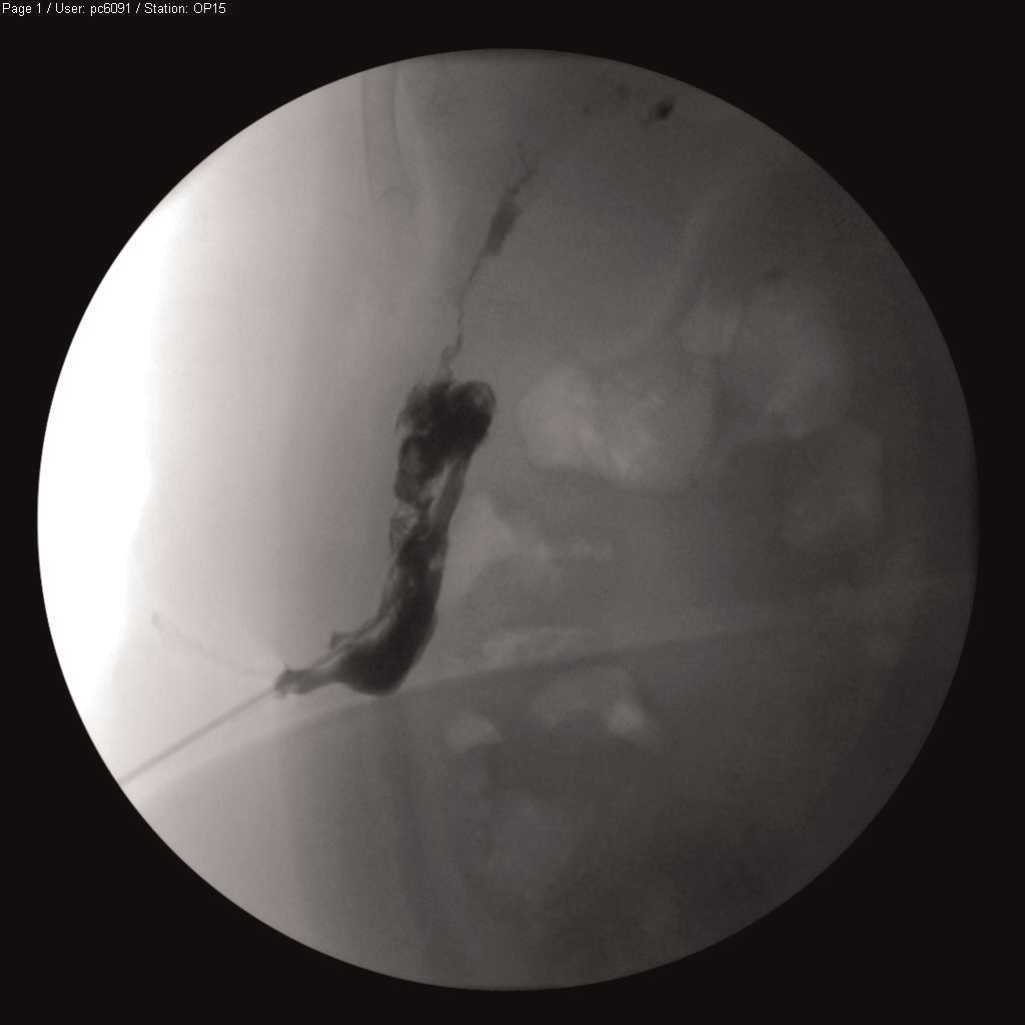
**Sinogram showing contrast running up the right paracolic gutter**.

Subsequently, the patient was treated as an out-patient, but her ultrasound scans (USS) continued to show collection of pus, which had to be drained three more times. The patient also developed chronic sinus discharge, and still went to the out-patient clinic 18 months after her initial presentation. A sinogram showed her sinus connecting with the right paracolic gutter and extending upwards and posteriorly (Figure 2). After identification of the offending gallstone on a second CT scan, the patient was offered surgery to remove the offending gallstones but declined this mode of treatment. At the time of writing she wished to continue with conservative management unless further problems arise.

## Discussion

We reviewed the published literature on spilled stones after laparoscopic cholecystectomy to discuss the risks, complications and management of patients who suffer from these lost stones.

### Risk of perforation of the gallbladder

Certain situations lead to higher risk of gallbladder perforation during laparoscopic cholecystectomy. Patients with acutely inflamed gallbladders have friable tissue which is susceptible to tear. Dense adhesions around the gallbladder make dissection potentially more difficult, and a tense, distended gallbladder that has not been decompressed is at risk of perforation [1,3]. This usually occurs when the gallbladder is manipulated by laparoscopic instruments or when it is dissected from the liver bed. Spilled stones are also caused by the slipping of the cystic duct clip or the tearing of the gallbladder while it is retrieved from the port site [4]. There is also a well recognised learning curve for performing laparoscopic cholecystectomies, and the risk of perforation is high early in a surgeon's laparoscopic career [1].

### Risk of complications from lost stones

Although lost gallstones were initially considered innocuous, it is now recognised that they can be a small but significant source of postoperative morbidity (0.1 to 6%) [4]. The presentation of complications will vary from patient to patient, and depend largely on the site and type of complication suffered. Recognised symptoms include abdominal pain, fever, abdominal masses, bowel obstruction and the presence of a sinus infection or fistula [2,5]. In some cases, the presenting mass has been diagnosed as malignancy until further investigations have disproved this. In most instances, the diagnosis is made retrospectively, or after visualisation of the stones on imaging and revisiting the patient's surgical history.

Most complications occur within the first few months, but presentations up to ten years after the procedure have also been documented [6]. Zehetner et al. looked into all documented complications from lost gallstones and these ranged from the most common like intra-abdominal and subcutaneous abscesses and fistulas, to the less common, such as liver abscess, staphylococcus bacteraemia, broncholithiasis and expectoration, empyema, granulomas, bowel obstruction and incarceration within a hernial sac [5].

Studies also show risk factors for complications after spilled stones, such as the presence of infected bile, spillage of pigmented gallstones, multiple stones (>15), stone size (>1.5 cm) and old age [5].

### Prevention and management of spilled stones

The best way to avoid complications from lost gallstones is to have awareness of the situations where perforation is likely, perform precise dissection, meticulously handle tissue and use devices such as endobags to retrieve dissected gallbladders through the port sites. Perforation usually occurs when dissecting the gallbladder from the hepatic fossa, and care taken at this stage of the operation can save many minutes attempting to retrieve stones from within the peritoneum [7].

Despite all precautionary measures, it is unavoidable that gallbladder perforation and stone spillage still occur in some patients. In these cases, it is crucial to minimise the number of stones spilled, attempt to retrieve all stray stones and to copiously irrigate the peritoneal cavity [4]. This serves the purpose of diluting any infected bile and may allow the stones to be washed up into the suction system. Some surgeons advocate the use of clips or an endoloop to close the hole in the gallbladder, while others will introduce a retrieval bag and 'park' it on the liver to receive all spilled stones [7]. In some situations it may be necessary to use an extra port adjusted to a 30- or 45-degree scope or use a fan liver retractor to improve visualisation [4].

Antibiotic prophylaxis is not routinely used by everyone, but its therapeutic use has been suggested for patients who undergo laparoscopic cholecystectomy to treat acute cholecystitis, have visibly infected bile, or have a high probability for lost stones. However, antibiotics should not be administered until the bile and stones have been collected for examination and culture, which would allow for the antibiotic selection to be tailored to the patient's condition [5].

Possibly the most important aspect in the management of perforated gallbladders and potential stone spillage is documentation. As already mentioned, diagnosis of complications related to lost stones is often done only after the identification of gallstones on radiological imaging. If the documentation is clear and the patient is aware of the perforation, then clinicians may be alerted early to the possibility of a stone complication in order to expedite treatment.

### Management of complications

The imaging method of choice is usually ultrasound, as stones are usually visualised well using this method. Visualisation, however, depends on the location of the lost stones. CT and magnetic resonance imaging (MRI) can also be used to obtain adjunct images depending on the biochemical composition of the stone. Radio-opaque calcified stones, such as pigmented stones, can be seen clearly on CT with unenhanced pictures. On MRI most stones are hypo-intense on T2-weighted images and iso-intense to hyperintense on T1-weighted images. These are best seen without fat suppression as this allows for the contrasting features of the stone to be seen against the fat [8]. Sometimes the radiological findings mimic unusual diagnoses such as actinomycosis, hydatid disease or even malignancy, so diagnosis can be difficult [1]. Ultimately, abscesses should be drained, whether percutaneously or surgically, and the stones should eventually be removed. Ideally this is done via minimally invasive techniques, but open surgery is often required. However, in our case, the patient was not keen on further invasive procedures and so for her the sequelae of lost stones may continue for years.

## Conclusions

Gallbladder perforation during laparoscopic cholecystectomy is a reasonably common problem and may result in spilled and lost gallstones. Though uncommon, these stones may lead to early or late complications, which can be a diagnostic challenge and cause significant morbidity to the patient. Proper care should be taken to avoid stone spillage. Should spillage occur, clear documentation and a high index of suspicion for complications should be maintained for early recognition and treatment of complications from this surgery.

## Abbreviations

CT: computerised tomography; MRI: magnetic resonance imaging; USS: ultrasound scan.

## Competing interests

The authors declare that they have no competing interests.

## Consent

Written informed consent was obtained from the patient for publication of this case report and any accompanying images. A copy of the written consent is available for review by the Editor-in-Chief of this journal.

## Authors' contributions

SH wrote the bulk of the manuscript and researched the literature. TS wrote some parts of the manuscript and also researched the literature. PS edited the final version.
